# Progression in immunotherapy for advanced prostate cancer

**DOI:** 10.3389/fonc.2023.1126752

**Published:** 2023-02-28

**Authors:** Hao Liang, Yang Liu, Jiao Guo, Maoyang Dou, Xiaoyi Zhang, Liyong Hu, Jun Chen

**Affiliations:** ^1^Department of Urology, Qilu Hospital of Shandong University, Jinan, Shandong, China; ^2^Department of Urology, Weifang People’s Hospital, Weifang Medical University, Weifang, Shandong, China; ^3^Department of Immunology, School of Basic Medical sciences, Weifang Medical University, Weifang, Shandong, China

**Keywords:** prostate cancer, immunotherapy, vaccine therapy, targeted therapy, combination therapy

## Abstract

Prostate cancer is one of the most common malignant cancers of the male genitourinary system and has high morbidity and mortality. Currently, treatment modalities for localized prostate cancer focus mainly on radical prostatectomy or radical radiation therapy. Some patients still experience disease recurrence or progression after these treatments, while others are already at an advanced stage or have metastases at the time of diagnosis. With the continuous development and progress of medicine in recent years, immunotherapy has become a revolutionary cancer treatment, and has achieved remarkable accomplishments in the treatment of hematologic malignancies. A variety of immunotherapies have also appeared in the field of advanced prostate cancer treatment, including therapeutic vaccines and immune checkpoint therapies. Despite the discrepancy between the results of some immunotherapy studies, immunotherapy for prostate cancer has shown some initial success, especially in combination immunotherapies. Currently, immunotherapy is mainly used in advanced prostate cancer, especially in patients with metastatic castration-resistant prostate cancer. However, with the development of more clinical trials of immunotherapy, more evidence will be provided supporting the rational application of immunotherapy in the future.

## 1 Introduction

Prostate cancer is one of the most common cancers among men worldwide, with an incidence of 1.4 million new cases per year. Approximately ten million men currently have prostate cancer worldwide, of which about 700,000 have metastasis, causing about 400,000 deaths each year (1, 2).

Current guidelines recommend radical prostatectomy or radiation therapy for early-stage localized prostate cancer (3–6). Some patients still experience disease recurrence or progression after treatment (7). For hormone-sensitive prostate cancer that responds to endocrine therapy, androgen-deprivation therapy (ADT) is typically maintained. Patients have a high response rate when they are initially treated with ADT, but long-term ADT leads to drug resistance. Androgen receptor (AR) amplification, AR mutation, AR splice variation, and the emergence of compensatory pathways are possible resistance mechanisms. Studies have shown that within 1–3 years of ADT, most patients experience progression of the cancer to metastatic castration-resistant prostate cancer (mCRPC), which defines patients who are at an advanced stage of the disease (8). The 5-year survival rate for patients with mCRPC is approximately 30% (9). Compared with conventional examinations such as CT and bone scan, the new PSMA-PET/CT and FDG-PET/CT have higher sensitivity for metastases, especially in patients with lower PSA levels (10–12). The advancement of imaging technology has further increased the number of mCRPC patients. Currently, a variety of drugs have been approved for the treatment of patients with mCRPC, such as the new generation of AR signaling inhibitors, chemotherapy drugs, bone-targeted therapy drugs, and poly-ADP-ribose polymerase (PARP) inhibitors (13). However, mCRPC remains an incurable fatal disease. In recent years, many new drugs have been approved for the treatment of hormone-sensitive prostate cancer (HSPC), and reports of cross-drug resistance in mCRPC patients have attracted wide attention (14), prompting us to explore a new, safer, and more effective cancer treatment. Immunotherapy for malignancies has achieved exciting results and a series of exploratory studies on immunotherapy for prostate cancer have been conducted. Immunotherapy enhances the immune system’s ability to recognize and kill cancer cells by regulating the autoimmune system, improving the antigen presentation ability, destroying the inhibitory tumor microenvironment, and reducing the apoptosis of effector cells to achieve the purpose of anti-tumor therapy (15–17). Prostate cancer has unique tumor characteristics compared to other tumors. First, it expresses multiple tumor-associated antigens: e.g., prostate-specific antigen (PSA), prostate-specific membrane antigen (PSMA), and prostate stem cell antigen (PSCA), which provide a reliable therapeutic target for prostate cancer immunotherapy (18–20). Second, the relatively “inert” tumor growth characteristics of prostate cancer also provide an extended window for cancer immunotherapy to establish an effective immune response. However, prostate cancer is a “cold” tumor that lacks immune cell infiltration (21). The low number of lymphocytes and the predominance of immunosuppressive components in the tumor microenvironment may limit the efficacy of immunotherapy (22, 23).

We review current research advances, clinical applications, and the risks and challenges related to prostate cancer immunotherapy. Most of these studies have been conducted in patients with advanced prostate cancer represented by mCRPC, so this will help us to understand some of the latest progress in the field of immunotherapy for advanced prostate cancer.

## 2 Therapeutic vaccine

There are various types of vaccines for prostate cancer treatment currently available, including cellular vaccines, viral vaccines, DNA vaccines, and other classifications, and in this section, we will present several representative vaccines.

Sipuleucel-T: first introduced in April 2010 and was the first therapeutic cancer vaccine approved by the Food and Drug Administration (FDA), primarily for use in asymptomatic or minimally symptomatic patients with mCRPC (24). The vaccine utilizes leukocyte isolation technology to isolate monocytes from the peripheral blood of the patient, which are cocultured *in vitro* with a recombinant fusion protein (PA2024) of prostatic acid phosphatase (PAP) and colony-stimulating factor (GM-CSF). granulocyte-macrophage. PA2024 stimulates the maturation of monocytes into dendritic cells that specifically present PAP. Dendritic cells activate PAP-specific cytotoxic T cells in patients after transfusion, enhancing their ability to recognize and kill prostate tumor cells. The results of the IMPACT phase III clinical trial (NCT00065442) demonstrated a survival benefit of Sipuleucel-T (25), compared to the placebo group, it prolonged the median overall survival (OS) of mCRPC patients by 4.1 months (median OS: 25.8 vs 21.7 months) and reduced the risk of death by 22%, hazard ratio (HR): 0.78, 95% CI: 0.61–0.98. This is consistent with another study showing an OS benefit of 4.5 months with Sipuleucel-T (26). However, there was no improvement in the time to disease progression. The study observed a more significant benefit in patients with low tumor load, suggesting a more significant OS benefit with early use of Sipuleucel-T in mCRPC patients. Similarly, an inverse association between PSA level and OS benefit was also seen in PROCEED study (27). Sipuleucel-T also exhibits a satisfactory safety profile, with studies reporting common adverse events (AEs) such as chills, fever, headache, muscle pain, and flu-like symptoms, which were associated with cytokine release after infusion. 65.2% of AEs were G1-G2, and most symptoms lasted no more than 2 days. Only 0.9% of the patients did not complete the infusion because of infusion-related adverse reactions (21). Several studies have recently been conducted to explore combination therapy regimens of Sipuleucel-T to analyze the most significant therapeutic benefit of Sipuleucel-T (28). It is still uncertain whether combination therapy can provide more benefit to specific groups, and we will introduce it in the subsequent combination therapy section of the article.

PROSTVAC: PROSTVAC is composed of a heterologous prime-boost regimen using two different live poxviral-based vectors: PROSTVAC-V, a recombinant vaccinia virus, and PROSTVAC-F, a recombinant fowlpox virus. The two vectors contain transgenes for human PSA and three costimulatory molecules (TRICOM: b7.1, LFA-3, ICAM-1). In phase II clinical trials (29), PROSTVAC prolonged median OS by 8.5 months and reduced the risk of death by 44% compared with placebo control, and corrected data expanded the survival benefit (median OS: 26.2 vs 16.3 months) and the survival advantage (HR=0.50) (30). Regarding safety, most AEs reported by PROSTVAC were local injection reactions, with fewer systemic AEs. The phase III PROSPECT trial compared patients treated with PROSTVAC+GMC-CSF, PROSTVAC alone, and placebo to further examine the effects of treatment (31). Contrary to the positive results of the phase II clinical trial, neither of the treatment groups effectively improved OS in the interim analysis, and the alive without events (AWE) rate was similar in both groups at six months. Events including radiographic progression, pain progression, initiation of chemotherapy for prostate cancer, or death, forced early termination of the trial. Regarding the differences in efficacy shown in the PROSPECT trial, an imbalanced allocation of prognostic-related factors in the phase II trial, may have amplified the benefits of OS in the treatment group; also, the smaller number of patients and possible observer bias may have affected the results. Additionally, including patients with multiple prior life-prolonging treatments in the PROSPECT trial may have influenced the positive outcome. Although the results of the phase 3 trial did not meet expectations, the PROSTVAC combination therapy study is still ongoing, and a study (NCT02933255) is exploring the safety and efficacy of PROSTVAC in combination with Nivolumab, and these combination therapies will provide more evidence on the appropriate use of PROSTVAC in the future (32, 33).

DCVAC/PCa is an active immunotherapy based on the activation of antitumor immunity by autologous dendritic cells. Dendritic cells are isolated from mononuclear cells in the peripheral blood of the patient by leukapheresis and brought into contact with dead human prostate adenocarcinoma cell lines, thus enhancing their antitumor activity. A single-arm phase I/II clinical trial in mCRPC patients confirmed that DCVAC/PCa combined with chemotherapy had a good safety profile. No serious adverse events (SAEs) related to DCVAC/PCa were reported in the study, and the median OS was 19 months, which was significantly improved compared with the predicted value of Halabi and MSKCC nomograms (34). Another study demonstrated that DCVAC/PCa produced durable immune responses and significantly prolonged PSA doubling time (PSADT) in prostate cancer patients with low tumor burden (35). The study also reported that the common AEs of DCVAC/PCa were local injection site reactions, fatigue, influenza like-illness, and mild infections, all of which were G1-G2. However, the VIABLE trial (NCT02111577) reported different results (36), with no significant OS benefit in the DCVAC/PCa combination chemotherapy group compared to the placebo group. No difference was observed in either of the primary efficacy endpoints. The VIABLE trial further provided good safety evidence for DCVAC/PCa, with most treatment-related AEs (TRAEs) associated with chemotherapy rather than DCVAC/PCa. The 119 patients who did not develop DCVAC/PCa were included in the efficacy analysis of the VIABLE trial. However, the shorter OS in this group of patients weakened the DCVAC/PCa treatment effect. Study found a dose-dependent treatment effect, with a subgroup of patients receiving more than ten doses of vaccine showing a propensity to benefit OS. Studies evaluating the efficacy of DCVAC/PCa in prostate cancer are still lacking, and more studies are needed to confirm its potential therapeutic value.

pTVG-HP[MVI-816] is a DNA vaccine that encodes the human PAP cDNA. pTVG-HP[MVI-816] has been previously studied for its favorable safety profile in patients with early PSA recurrent prostate cancer, and enhanced vaccine-induced PAP-specific Th1 cell responses have been observed (37). There are no reports on the efficacy and safety of the pTVG-HP[MVI-816] vaccine alone in large trials in patients with mCRPC. In the phase II clinical trial of non-metastatic hormone-sensitive prostate cancer (nmHSPC) with biochemical recurrence (38), there was no significant difference in 2-year metastasis-free survival (MFS) in the pTVG-HP[MVI-816] group (41.8% vs 42.3% P=0.97). Regarding secondary endpoints, no significant differences were observed between the two groups in median MFS and median PSADT; partial immune responses were observed early in treatment but then disappeared. Difficulty in maintaining long-term immune responses may be the main obstacle limiting the antitumor efficacy of pTVG-HP. There is no substantial evidence to support that a single regimen of MVI-816 may make a meaningful difference for patients, and we are counting on whether a combination regimen can enhance its efficacy. A recently published study comparing the effectiveness of MVI-816 in combination with pembrolizumab in patients with mCRPC reported a preliminary exploration of the optimal dosing regimen for combination therapy. The results showed that the combination therapy was superior to PD-1 or PD-L1 monotherapy in PSA declines, tumor volume decreases, and 6-months DCR, while the combination therapy had a good safety profile. G2 or higher TRAEs occurred in 42% of the patients, and common TRAEs were thyroid dysfunction, adrenal insufficiency, colitis, and hepatitis (39). Another DNA vaccine, pTVG-AR [MVI-118], which contains cDNA encoding the ARligand-binding domain (AR-LBD), has been evaluated in a completed multicenter phase Ι trial (NCT02411786) and showed a favorable safety profile and durable immune responsiveness (40).

In summary, durable immune responses specific to tumor antigens have been observed in studies of multiple prostate cancer therapeutic vaccines. However, there is still a lack of consistent clinical evidence confirming the therapeutic efficacy of vaccines, except for Sipuleucel-T, for which several recent large trials have provided conflicting results. Vaccine combination therapy appears to have gained more attention in recent years, which may provide new approaches for subsequent treatment and provide a rationale guiding and supporting the exploration and use of prostate cancer vaccine therapy.

## 3 Immune checkpoint therapy

There are antagonistic mechanisms of promotion and suppression of the immune system in the development of tumors: Conversely, when some activating signals stimulate cytotoxic cells with tumor-killing capacity, they will promote functional phenotype transformation and accelerate cell proliferation, which can enhance their ability to kill cancer cells. Conversely, when cytotoxic cells are stimulated by inhibiting signals from the surrounding environment, they will cause their dysfunction and inhibit their proliferation, thus weakening their ability to kill tumor cells. Cells exhibit negative regulatory function *via* a receptor or ligand called an immune checkpoint, and anti-tumor therapy targeting the regulating of immune checkpoints is called Immune Checkpoint Therapy (ICT) (41, 42). Food And Drug Administration (FDA) has approved ICT for the treatment of solid malignancies in multiple organs (43–45). The main therapeutic targets of ICT in prostate cancer are the cytotoxic T lymphocyte-associated antigen 4 (CTLA-4), programmed cell death protein 1 (PD-1) and its ligand (PD-L1) (46, 47).

Ipilimumab is a humanized monoclonal antibody that blocks CTLA-4 and enhances the immune effect of T cells. It was approved in 2011 for the treatment of melanoma (48). Some early clinical trials that confirmed the anti-tumor activity of Ipilimumab in solid tumors included patients with prostate cancer. However, the results of a phase III trial in patients with asymptomatic or minimally symptomatic mCRPC without visceral metastases who had not previously been treated with chemotherapy (49), did not show a benefit in OS following treatment with ipilimumab compared to placebo. In a separate phase III study (50), the ipilimumab group did not show any significant OS benefit compared with placebo in a population of minimally symptomatic mCRPC patients who had received prior docetaxel chemotherapy and were chemotherapy-sensitive, despite the presence of long-term responders. However, we found that a small group of patients in this study achieved a significant and sustained clinical response with ipilimumab. A follow-up study found that a subgroup of patients with mCRPC with immune characteristics such as higher intratumor infiltrating CD8+ T cells, high IFN-γ response gene signals, and more robust antigen-specific T cell responses was more likely to achieve control of progression with ipilimumab monotherapy and more extended survival benefits, despite the relatively low tumor mutational load in this subset of patients (51). This finding indicates that a more careful selection of appropriate patients is required for ipilimumab treatment.

PD-1 is expressed in activated T cells and binds to PD-L1 and PD-L2 to mediate inhibition of the activity of variable tumor effector cells (52). In patients with metastatic melanoma, objective response rates (ORR) ranging from 20% to 45% were observed after CTLA-4 or PD-1 (53), with response rates up to 60% observed when CTLA-4 was combined with PD-1 blockade (54, 55). However, similar to ipilimumab monotherapy, studies have found that nivolumab and pembrolizumab alone do not achieve the expected treatment outcomes in patients with advanced prostate cancer (56–58). In the KEYNOTE-199 trial (58), the ORR (5% vs 3%) and disease control rate (DCR) (13% vs 18%) in PD-L1-positive patients were similar to the PD-L1-negative patient group; no differences in OS were observed between the two groups, which may be related to the more advanced stage of the disease in the positive PD-L1 group. It is believed that the immunosuppressive tumor microenvironment, tumor mutation burden and immune escape mechanism of prostate cancer are the reasons that hinder the efficacy of immune checkpoint inhibitors (59). At the same time, the shorter duration of treatment in KEYNOTE-199 could diminish the OS benefit. Previous studies have found that ipilimumab treatment significantly increased the number of tumor-infiltrating T cells in prostate cancer patients. However, it induced a compensatory immunosuppressive pathway mediated by PD-1/PD-L1 signaling, negatively affecting antitumor therapy (60). Based on this finding, the subsequent CheckMate650 Phase II trial (NCT02985957) focused on improving treatment outcomes for patients with prostate cancer when ipilimumab was administered in combination with nivolumab (61). We will describe this study in more detail below with regard to the combination therapy strategies. The European Association of Urology (EAU) guidelines suggest that pembrolizumab may be a valuable additional management strategy for mCRPC patients with high microsatellite instability, and with continuous advances in genome sequencing technology, it will be helpful to screen patients who can benefit from immunosuppressive therapy (62, 63).

The difficulty of producing substantial clinical benefits with ICT alone in unselected patients with prostate cancer has been widely recognized. However, earlier studies have reported that a subgroup of prostate cancer patients with defects in the DNA mismatch repair gene and high microsatellite instability characteristics tended to have higher response rates to single ICT (64, 65). The results of the subgroup analysis also suggested that single immunological checkpoint blockade had better efficacy in these patients. However, this idea has been questioned in recent studies: it has been shown that there is no positive correlation between CD8+ T cell numbers and neoantigen load in breast and prostate cancers and that the characteristics of high tumor mutational load is not predictive of the efficacy of ICT in patients with breast and prostate cancer. Therefore, the search for additional biomarkers as predictors of immune checkpoint efficacy may be required in the future (66, 67). This highlights the importance of tumor signature screening for prostate cancer patients and individualized treatment regimens for prostate cancer in terms of immunotherapy strategies.

## 4 Adoptive cell therapy

Adoptive cell therapy is a rapidly expanding field of medicine in recent years that mediates antitumor, antiviral, or anti-inflammatory effects by isolating, modifying, and expanding autologous or allogeneic tumor-responsive lymphocytes and reinfusing processed lymphocytes back into the patient (68, 69). Of these, cell therapies involving chimeric antigen receptor T (CAR-T) have demonstrated high response rates and durable disease remission in the treatment of hematologic malignancies (70–72), and several companies have received FDA approval for their CAR-T products for the treatment of refractory and complex hematologic diseases in the last 5 years (73).

CAR-T therapy genetically modifies T cells by *in vitro* transfection technology to express engineered chimeric receptors (74). Currently, CAR-T technology has developed to the fourth generation, as the latest generation technology, the structure not only has a co-stimulatory protein intracellular domain but also promotes the release of cytokines such as IL-12, IL15, and IL18 after receptor activation, which can enhance the killing efficiency of T cells (75). When CAR-T cells are cultured and expanded *in vitro* and transfused back to patients, the transmembrane region converts the CAR recognition signal for extracellular targeted tumor antigens into a signal for activation of T cells through the intracellular domain. When CAR-T cells arrive inside the tumor, they cause cytotoxic particles such as cytokines and perforins to be secreted by cytotoxic T cells, leading to the destruction of tumor cells (76). This technique is being applied to treat solid malignancies, including prostate cancer (77, 78). This is a new T cell-mediated antitumor therapy in which cytotoxic T cells can be activated independently of major histocompatibility complex (MHC), thus eliminating dependence on the traditional T cell receptor-MHC pathway, can be severely compromised in the “cold” tumor microenvironment of prostate cancer (79).

Narayan et al. reported the results of the latest phase I clinical trial of CAR-T therapy in patients with mCRPC (80): In terms of safety, a cytokine release syndrome (CRS) was the most common drug-related SAEs, predominantly G1-3, and most patients exhibiting a CRS resolved spontaneously or with symptomatic treatment, confirming its good safety profile. In terms of tumor responsiveness, PSA levels decreased by at least 30% in 4 of the 13 patients; one patient had a >98% decrease in PSA levels accompanied by significant proliferation of CAR-T cells *in vivo*, and 38.5% of patients maintained stable disease status at three months posttreatment assessed by imaging. The study showed a median OS of 15.9 months and a median progression free survival (PFS) of 4.4 months in patients receiving CAR-T therapy. Although a general immune response was observed in the study, it does not seem to translate into a survival benefit for mCRPC patients. The study also observed that patients had a dose-dependent decrease in peripheral blood CAR-T cell proliferation, inflammatory cytokine expression, clinical CRS, and PSA, which could help guide the appropriate dose selection for future CAR-T therapies in clinical applications (81). This result is generally consistent with the results of the earlier P-PSMA-101-001 trial (NCT04249947). Currently, there is no consensus on whether to receive lymphatic clearance prior to CAR-T therapy, and studies have shown that lymphatic clearance enhances T cell proliferation and viability, thus improving efficacy but also increasing hematologic and systemic toxicity. Thus, more research is needed to select suitable patients to receive lymphatic clearance to achieve maximum therapeutic benefit.

Although CAR-T therapy has shown good therapeutic potential in the treatment of advanced prostate cancer, several barriers remain to be addressed to enhance CAR-T therapy efficacy in solid tumors: 1) physical interference of CAR-T cells by the stroma surrounding solid tumors; 2) abnormal CAR-T function due to the specific suppressive tumor microenvironment of prostate cancer; and 3) CAR-T cell defects: reduced self-replication ability (82, 83). Multiple studies of CAR-T therapies are ongoing (NCT03873805; NCT02744287). A preclinical study has shown that CAR-T combined with docetaxel has synergistic efficacy (84), and this feasibility needs to be supported by evidence in future clinical trials. With the improvement of the structure of CAR-T cells, as well as the determination of therapeutic dose and treatment cycle, CAR-T may become an alternative treatment for prostate cancer patients.

## 5 Bispecific antibody therapies

Bispecific antibody therapies, especially bispecific T-cell engagers (BiTE) (85), have shown significant therapeutic promise in the treatment of refractory hematologic malignancies. Recently, BiTE therapies have been explored to treat advanced malignant solid tumors. The studies conducted to date have mainly included patients with mCRPC. BiTEs utilize single chain variable fragment (ScFv) technology to recognize specific tumor antigens. Antibodies on one side of the BiTE bind specifically to tumor cell surface tumor antigens, such as PSMA, generating activation signals delivered to the T cell CD3 surface receptors *via* antibodies on the other side (86). Direct engagement of co-stimulatory CD3 receptors bypasses the need for traditional monosynaptic binding and enables MHC non-dependent T cell activation. In recent preclinical studies (87), AMG160 showed promising durable specific antitumor activity and an acceptable nonclinical safety profile in a model of prostate cancer tumor graft. There is a lack of solid evidence supporting the clinical application of BiTE in large trials, with safety and efficacy results of BiTE in patients with prostate cancer reported only in phase I clinical trials (88). In terms of imaging, there is evidence that AMG160 does not interfere with the signal intensity of 68Ga-PSMA-11PET/CT compared to non-PSMA specific BiTE, which has important implications for the post-treatment efficacy assessment (89). BiTE is more readily available for widespread use than CAR-T therapy because it is not a separately produced cellular product. In terms of tumor penetration capacity, BiTE therapy is superior to CAR-T, and in terms of safety, the incidence of BiTE adverse events is lower and relatively controllable. However, there are still many challenges for BiTE therapy, such as loss of target antigen, formation of resistant antibodies, and up-regulation of immune checkpoints (90, 91). The up-regulation of immune checkpoint is a possible resistance mechanism of BiTE, which provides theoretical support for the combination of BiTE therapy and ICI (92). In addition to targeting PSMA, exploring other alternative tumor antigens, such as PSCA, disintegrin and metalloproteinase 17 (ADAM17M) and delta-like ligand 3 (DDL3), may also be a future direction (93–95).

## 6 Combination therapy

Current evidence shows that treatment with single immunotherapy regimens appear have not achieved the expected therapeutic effects. With the increasing understanding of the regulatory mechanisms of immunotherapy in various preclinical studies, immunotherapy-based combination therapy strategies are gradually becoming and increasing trend. Current combination treatment options include the combination of multiple immunotherapy regimens, immunotherapy combined with hormone therapy, immunotherapy combined with radiation therapy, and immunotherapy combined with chemotherapy.

### 6.1 Immune dual combination therapy

Uncertainty about the efficacy of immune checkpoint therapy monotherapy regimens facilitated the exploration of combination regimens, and the establishment of the CheckMate650 phase II trial (NCT02985957) (61). The no-chemotherapy cohort and the post-chemotherapy cohort received ipilimumab (3 mg/kg) in combination with nivolumab (1 mg/kg) with a median follow-up of 11.9 months and 13.5 months, respectively, ORR of 25% and 10% in the two groups, and median OS of 19.0 months and 15.2 months, respectively. Although combination therapy demonstrated significant treatment effects, the study reported a significantly increased incidence of TRAEs, with approximately 40% of patients in both groups requiring the application of high-dose cortisol for immune-mediated AEs. Approximately half of the patients exhibited G3-G4-grade TRAEs and 4 patients experienced TRAE-related deaths. Higher drug-related mortality in patients with mCRPC compared to the same dose regimen previously applied in patients with metastatic melanoma may be associated with advanced age and worse ECOG scores. The significant treatment effect observed in the CheckMate650 trial revealed the therapeutic promise of dual immunosuppressant combinations, and a concomitant increase in drug toxicity needs to be investigated in future studies. We must explore different dosing strategies to find the balance between efficacy and toxicity. The other study investigated the combination of Sipuleucel-T and Ipilimumab in patients with mCRPC, and the combination did not achieve greater efficacy than Ipilimumab monotherapy, which is similar to the results of the previous study (96, 97). It was found that the timing of Ipilimumab administration after Sipuleucel-T vaccination may affect the activation of antigen-specific T cells, but no differences in patient survival benefit and disease progression were observed. Similarly, no additional benefit was shown with Sipuleucel-T plus pTVG-HP as an immune-boosting regimen (98).

### 6.2 Immunotherapy combined with hormone therapy

Hormone therapy plays an important role in the treatment strategy of prostate cancer patients, and the new generation of AR pathway inhibitors provide more options to treat patients with advanced prostate cancer (99, 100). In recent years, as the mechanisms of androgen activity in prostate cancer have been studied, we have gained a richer understanding of the immune regulatory mechanisms played by androgens and AR in patients with prostate cancer. AR is expressed not only in tumor cells but also in various immune cells *in vivo*, playing an immunomodulatory role (101, 102). Androgens have long been known to inhibit the development and activation of T and B cells through multiple mechanisms (103). In patients with prostate cancer, immunotherapy combined with hormone therapy has emerged as a new combination therapy. However, the efficacy of immunotherapy combined with hormone therapy in patients with prostate cancer has produced uncertain findings. Conflicting treatment outcomes are usually attributed to differences in patient populations. Recent studies have found that AR antagonists interfere with initial T cell activation and may diminish the therapeutic effect of combination therapy (104). However, this immunosuppressive effect can be avoided by judicious selection of the sequential dose timing (105, 106). A recent study published in Nature revealed a potential mechanism by which AR antagonists in combination with anti-PD-1 monoclonal antibodies in clinical trials led to high patient responsiveness (105). The study reported that enzalutamide prevented T cell depletion by inhibiting AR in CD8+ T cells while increasing IFN-γ release and improving responsiveness to targeted PD-1 therapy, which provided a theoretical basis for the administration of hormone therapy in combination with immune checkpoint inhibitors. The IMbassador250 trial (NCT03016312) investigated the impact of co-administration of atezolizumab with enzalutamide compared to enzalutamide alone on the survival benefit of patients with mCRPC (107). Although the incidence of AEs in the combination group was essentially identical to enzalutamide alone, the combination group (median OS: 15.2 months) did not show a survival benefit compared to enzalutamide alone (median OS: 16.6 months) (HR=1.12 95% CI 0.91–1.37), forcing the early termination of the study. In terms of secondary outcomes, the combination group similarly did not show any benefit. It is difficult to provide a plausible explanation for the IMbassador250 trial results. However, previous single-arm studies have found that pembrolizumab combined with enzalutamide produced an 18% response rate in unselected mCRPC. In the latest Nature study, the ADT+enzalutamide+anti-PD-L1 triplet regimen provided a superior OS benefit and the most significant reduction in tumor volume in prostate cancer and sarcoma models compared to the duplex regimen, and it appears that ADT enhanced the synergistic effect of enzalutamide in combination with immunotherapy. A clinical trial of triple combination therapy ADT + enzalutamide + pembrolizumab (NCT04191096) is underway in patients with mHSPC, which will further validate the safety and patient responsiveness of the triple combination regimen. We cannot help but look forward to the therapeutic potential of the triple combination regimen for advanced prostate cancer.

### 6.3 Immunotherapy combined with radiation therapy

Radiation therapy has been one of the practical tools for the treatment of various malignant tumors, and it can stimulate the production of tumor-specific immune responses by inducing tumor cell death, enhancing the release of tumor-associated antigens, and upregulating the expression of tumor suppressor proteins and cytokines through various pathways and mechanisms (107, 108). In recent years, targeted radiotherapy, represented by Ra-223, has gradually gained clinical popularity as an emerging therapeutic tool for patients with advanced prostate cancer, especially for those with combined bone metastases (109). Ra-223 is a radioactive calcium analogue that selectively binds to areas of increased bone transformation and has a certain degree of “bone targeting”. It produces antitumor effects by releasing alpha particles into surrounding tissues to destroy cellular DNA. Due to the small diameter of the action of the alpha particle (2–10 cell diameters), Ra-233 causes less damage to surrounding normal tissues, giving it a better safety profile (110). In 2013, Ra-223 was approved by the FDA for the treatment of patients with symptomatic mCRPC with bone metastases (111). The survival benefit and the improvement of bone-related events in mCRPC patients have been supported by the results of several large clinical trials, in which Ra-223 significantly prolonged OS and PFS in patients with advanced prostate cancer, reduced bone pain symptoms considerably, and delayed the onset of bone-related events during treatment (112, 113). Several trials have recently explored the feasibility of a combination immunotherapy regimen with Ra-223. A recently completed trial of Sipuleucel-T in combination with Ra-223 demonstrated superiority to Sipuleucel-T administered alone in patients with mCRPC with bone metastases (114). The study found that Sipuleucel-T in combination with Ra-223 did not increase the incidence or severity of adverse events; however, the combination group did not demonstrate an advantage in secondary outcome indicators related to the immune response. The combination group showed higher PSA responsiveness (33% vs 0%) and longer time to tumor progression in the observation of patient clinical outcomes (median PFS 39w vs 12w; HR=0.32), which was consistent with the findings obtained in previous studies (115), in which Sipuleucel-T combined with Ra-223 produced a more significant benefit in patients with mCRPC without additional toxicity. However, not all combination therapies with Ra-223 produced exciting results; for example, trials exploring the administration of atezolizumab in combination with Ra-223 did not produce any additional therapeutic benefits in the combination group but instead resulted in more significant drug toxicity in the combination group compared to monotherapy, Thus, combination therapy with Ra-223 needs to be further studied (116).

Another radiopharmaceutical with great therapeutic potential, 177Lu-PSMA-617, specifically identifies tumor cells with high expression of PSMA and releases β-particles to destroy tumor cells, was recently evaluated in the just concluded VISION trial (117). Significant benefits in radiology progression-free survival (rPFS) (8.7 vs 3.4 months, HR=0.40) and OS (15.3 vs 11.3 months, HR=0.62) have been reported, and significant improvements were also observed in all secondary endpoints of the study. Because 177Lu-PSMA-617 also has a favorable safety profile, it has been described as a revolutionary precision radiotherapy modality for the treatment of mCRPC. The PSA response rate was superior to that reported for cabazitaxel and docetaxel in other studies (118, 119). The efficacy and safety of 177Lu-PSMA-617 combined with immunotherapy or other drugs have been explored in patients with mCRPC. Considering the low possibility of overlap in toxicity of radiotherapy combined with other therapeutic agents, 177Lu-PSMA-617 combination therapy may provide a relatively safe treatment option for patients with mCRPC.

### 6.4 Immunotherapy combined with chemotherapy

Chemotherapy, a conventional treatment for cancer patients, is widely used in the treatment of various malignant diseases. Docetaxel and cabazitaxel have been successively approved for the treatment of patients with mCRPC and have been shown to prolong patient survival and control disease progression. Some studies have shown that chemotherapy-induced tumor cell destruction may enhance the development of specific immune responses (120). A study evaluating pembrolizumab in combination with docetaxel for mCRPC reported the efficacy of combination therapy in patients with mCRPC previously treated with enzalutamide or abiraterone. The PSA response rate was 27% and ORR and DCR were 23% and 52%, respectively. The median OS of combination therapy was 20.2 months, which was significantly prolonged compared to the median OS of 9.5 months in the PD-L1 positive cohort in KEYNOTE-199. KEYNOTE-365 showed initial success with PD-L1 antibodies in combination with chemotherapy. However, the patients included in the study were previously chemotherapy naïve patients. All KEYNOTE-199 patients were treated with docetaxel chemotherapy prior to treatment with PD-L1 antibodies. Nonetheless, we cannot define the specific impact of chemotherapy on the benefit of combination therapy. The ongoing KEYNOTE-921 trial includes patients with mCRPC after chemotherapy and will provide further evidence to support this combination strategy (121).

### 6.5 Immunotherapy combined with PARP inhibitors

PARP inhibitors have recently become one of the most popular drugs in the mCRPC therapeutic area, and is represented by olaparib and rucaparib. PARP plays an important role in DNA damage repair *in vivo*, and PARP1 and PARP2 mediate DNA damage repair through base excision. The restoration of single-stranded DNA (ssDNA) damage can be blocked by PARP inhibitors. Homologous recombination repair proteins can compensate for the above by repairing broken double-stranded DNA. However, under the homologous recombination repair gene defect (HRD), this compensatory pathway is blocked. PARP inhibitors and HRD cause a synthetic lethality of tumor cells, generating tumor neoantigens that increase immunogenicity and improve immune responsiveness in the tumor microenvironment (122). The safety and significant therapeutic effects of olaparib and rucaparib monotherapy in patients with mCRPC have been reported in several studies (123, 124). In the TOPARP-A trial, the ORR to treatment was 32%, with a response rate of 88% in patients with DNA repair gene mutations, and other studies have confirmed that patients with genetic defects such as BRCA1, BRCA2, ATM, FANC, and CHEK2 have higher sensitivity to PARP inhibitors (125). PARP inhibitors have been shown to have synergistic effects with PD-1/PD-L1 or CTLA-4 blockade (126, 127). A recent study of rucaparib in combination with nivolumab for mCRPC reported its results: regardless of previous chemotherapy (128), the CheckMate 9KD study showed significantly improved ORR and PSA response rates with combination therapy in the HRD+ cohort, particularly in patients with BRAC1/2 mutations; in terms of OS and rPFS, the median OS for the A2 cohort without chemotherapy was 20.2 months (95% CI 14.1–22.8 months) and the median rPFS was 8.1 months (95% CI 5.6–10.9 months). Regarding safety, common TRAEs observed following combination therapy were nausea, fatigue, anemia, and loss of appetite, with G3-G4 TRAEs occurring in half of the patients in both cohorts, and neutropenia warranting focus during treatment. In general, rucaparib combined with nivolumab did not appear to show additional benefits in unselected mCRPC patients, which is consistent with the findings of the previous KEYLYNK-010 trial of olaparib combined with pembrolizumab. It is also encouraging that a significant response to combination therapy was observed in the subgroup of patients with BRAC1 and BRAC2 mutations, but the failure to translate into a survival benefit is difficult to explain and will require further evidence. Many studies have been conducted to screen and identify new, highly effective therapeutic predictive factors for mCRPC patients (129). Continued advances in next-generation exon sequencing technology and liquid biopsy technology will contribute to the precise treatment of mCRPC patients and will provide more significant benefits to patient.

### 6.6 Immunotherapy combined tyrosine kinase inhibitors (TKIs)

TKIs represented by Cabozantinib and Masitinib belong to the class of small-molecule inhibitors with RARP inhibitors, and both drugs have shown antitumor activity in previous studies. Cabozantinib is a mesenchymal-epithelial transition factor (c-MET) and vascular endothelial factor receptor 2 (VEGFR2) inhibitor that has been approved for the treatment of patients with advanced renal cell carcinoma. In a phase 2 study, cabozantinib significantly prolonged PFS in patients with CRPC (130). However, in the COMET-1 study, mCRPC patients after chemotherapy failed to show an OS benefit (131). Studies have shown that Cabozantinib has immunomodulatory effects and may be synergistic with other immunotherapy combinations (132, 133). The COSMIC 021 trial evaluated the safety and clinical benefit of Cabozantinib in combination with atezolizumab in patients with mCRPC. The study found that the combination regimen had better PSA response rate and DCR than either drug monotherapy. In terms of safety, 95% of patients experienced TRAEs at any grade and 55% of patients experienced G3-G4. The most common G3-G4 AEs were pulmonary embolism, diarrhea, fatigue, and hypertension. The safety profile of cabozantinib combined with atezolizumab was generally consistent with that of the individual agents, but the incidence of pulmonary embolism is higher than that of monotherapy (134). Elderly age and concurrent use of ADT are possible causes of pulmonary embolism. Due to the differences in patient groups included in different studies, it is difficult to compare the specific benefits of combination therapy at present, and future evidence support from other studies is needed.

## 7 Limitations and future prospects

There are still many obstacles and challenges in prostate cancer immunotherapy, such as the balance between efficacy and toxicity of immunotherapy, the timing of sequential administration, the requirement of individualized dosing regimen for prostate cancer due to tumor heterogeneity, the lack of appropriate biomarkers for efficacy evaluation, and the insufficient understanding of the mechanism of drug resistance. These issues need to be focused on in the future. Due to the extensive differences between the studies of immunotherapy, there is a lack of direct evidence to support the comparison of the treatment effects of different regimens, and more large-scale controlled trials are needed in the future. Immunotherapy of prostate cancer is a promising treatment. With the deepening of research, new tumor-specific antigens have been discovered, which provides more potential targets for immunotherapy. The continuous progress of high-throughput sequencing technology and liquid biopsy technology has promoted the identification of tumor heterogeneity of prostate cancer and promoted the precision treatment of prostate cancer patients. The continuous exploration of drug combination is helpful to the study of drug interaction mechanism. The development of imaging technology represented by PSMA-PET/CT provides a powerful aid in disease diagnosis and efficacy evaluation.

## 8 Conclusion

Over the past decade, as immunotherapy for solid tumors continues to be explored, our understanding of immunotherapy and immunomodulation of solid tumors, including prostate cancer, has also improved, and a variety of immunotherapeutic agents, including tumor vaccines and immune checkpoint inhibitors, have achieved exciting results in clinical trials for advanced prostate cancer. Although most current trials on immunotherapy for prostate cancer have focused on patients with mCRPC, there is reason to believe that immunotherapy may bring earlier clinical benefits to prostate cancer patients as immunotherapy continues to improve and mature. In this review, we summarize the experience and lessons learnt from recent immunotherapy studies and update the theoretical basis and regulatory mechanisms underlying immunotherapy for prostate cancer, helping to understand the latest progress in immunotherapy for prostate cancer. As more and more clinical trials are conducted, these will provide strong evidence to support and compare the efficacy of immunotherapy for prostate cancer, providing a valuable reference that will allow more patients with prostate cancer to choose their treatment regimen and prolong survival and also improve the quality of patient survival.

## Author contributions

HL and YL wrote the first draft of the manuscript. YL, JG, MD, and HL wrote sections of the manuscript. XZ and LH polished the language and searched the literature. All authors contributed to the article and approved the submitted version.
